# Leukocyte Biomarkers for the Differential Diagnosis of Mild Acute Ischemic Stroke, Transient Ischemic Attack, and Stroke Mimic

**DOI:** 10.7759/cureus.13383

**Published:** 2021-02-16

**Authors:** Megan E Cavrak, Rotem Hass, Ronald J Stephens, Amelia Adcock, Ashley B Petrone

**Affiliations:** 1 Pathology, Anatomy and Laboratory Medicine, West Virginia University School of Medicine, Morgantown, USA; 2 Neurology, West Virginia University School of Medicine, Morgantown, USA

**Keywords:** stroke, biomarker, immune, neutrophil, leukocyte, transient ischemic attack (tia), mild acute ischemic stroke (ais)

## Abstract

Introduction

The differential diagnosis of transient ischemic attack (TIA) versus mild acute ischemic stroke (AIS) during the initial presentation to the emergency department is often difficult, as the diagnosis of both TIA and AIS relies on the presence of focal neurologic signs. As such, roughly 50% of patients with transient or mild neurologic deficits have an uncertain diagnosis prior to neuroimaging. Biomarkers, particularly leukocyte biomarkers, may be used by clinicians to diagnose mild AIS prior to neuroimaging, and this study is the first to describe the use of leukocyte biomarkers for the differentiation of mild AIS, TIA, and stroke mimic.

Methods

We performed a retrospective chart review of patients discharged from a local hospital with a discharge diagnosis of either TIA or AIS. Past medical history and complete blood cell count with differential upon admission were collected for all subjects. Statistical analyses were performed to compare immune cell parameters between the two groups. For all comparisons, logistic regression analysis was used to assess the effect of confounding variables, such as age, gender, and medical history for each study variable.

Results

Of all the immune parameters assessed in this study, the neutrophil percentage was the only significant biomarker that significantly differed between study groups. After adjustment for confounding variables using stepwise logistic regression, mild AIS patients were 5.3 times more likely than TIA cases to have a neutrophil percentage above the normal range.

Conclusion

Our results suggest that clinicians may utilize neutrophil percentage as an additional piece of information that may aid in their diagnosis of mild AIS versus TIA.

## Introduction

A transient ischemic attack (TIA) is a clinical syndrome associated with a transient, regional reduction in cerebral blood flow, leading to a sudden onset of rapidly resolving focal neurologic deficits [1]. The duration of deficits associated with TIA are variable; however, TIA has been broadly defined as a brief episode of focal neurological deficits lasting less than 24 hours, although typically resolve within minutes. Regardless of the duration of symptoms, in contrast to acute ischemic stroke (AIS), TIA does not result in an ischemic infarct. As such, it is often difficult to differentiate TIA from mild AIS during the initial presentation to the emergency department, as the diagnosis of both TIA and AIS relies on the presence of focal neurologic signs. Due to this, approximately 50% of patients presenting to emergency departments in North America with transient or mild neurologic deficits have an uncertain diagnosis and prognosis prior to neuroimaging [1]. In a typical stroke imaging protocol, patients receive a non-contrast computed tomography (CT) scan to potentially confirm the presence of an infarct but, more importantly, to rule out an intracranial hemorrhage in order to administer tissue plasminogen activator (tPA). While this CT is rapid and appropriate for tPA decision-making, in a majority of cases, magnetic resonance imaging (MRI) is necessary to confirm AIS, as 60% to 70% of AIS cases, later confirmed with MRI, are initially CT-negative [1]. While an MRI may be ordered after the acute treatment period to confirm AIS, the 2019 American Heart Association Guidelines for the Management of Acute Ischemic Stroke Care do not suggest that MRI should be used in acute decision-making nor is it a requirement for AIS diagnosis [2]. Thus, without MRI, the differential diagnosis between mild AIS, TIA, and stroke mimic can be both difficult and subjective to clinicians. However, reliance on MRI increases or delays the time to diagnosis. 

One potential solution to this problem is the utilization of a rapidly obtainable diagnostic biomarker. We and numerous others have previously shown that immune biomarkers may be useful in the diagnosis of AIS, but a limited number of studies have investigated immune biomarkers for the differential diagnosis of AIS vs. TIA vs. stroke mimic. Furthermore, several of these studies have combined AIS and TIA into a single group for comparison. For example, Ross et al. and Lim et al. compared immune cell counts between AIS and TIA versus control, and while both studies reported significantly increased neutrophil percentage and decreased lymphocyte percentage in AIS and TIA compared to control, neither of these studies compared AIS to control [3-4]. Gökhan et al. reported that the neutrophil count and neutrophil-lymphocyte ratio (NLR) were significantly higher in AIS compared to TIA [5]. However, the authors did not include a control or a stroke mimic group for comparison, nor was the National Institutes of Health Stroke Scale (NIHSS) reported to determine the severity of the AIS patient sample. Celikbelik et al. reported that NLR was significantly higher in AIS and TIA versus control; however, there was no reported difference between AIS vs. TIA nor TIA vs. control [6]. Armstrong et al. reported that the neutrophil count was significantly higher in AIS compared to TIA, but not TIA compared to control [7]. However, the mean NIHSS stroke score was 6 (range: 4 - 19); therefore, the AIS group included moderate to severe cases.

As such, no single study has included a stroke mimic group for comparison nor has a study described the accuracy in immune biomarkers in the scenario that is often most difficult for clinicians, which is differentiating stroke mimic vs. TIA vs. mild AIS. Thus, the overall purpose of this study was to evaluate the accuracy of immune biomarkers, particularly neutrophils, in the differential diagnosis between stroke mimic, TIA, and mild AIS.

## Materials and methods

This study received institutional review board approval from West Virginia University. We performed a retrospective chart review of patients discharged from a local hospital with a discharge diagnosis of either TIA (ICD9- 435.9: ICD10- G45.9) or AIS (ICD9-434: ICD10- I63). In addition to the discharge diagnosis, TIA patients had to meet several study inclusion criteria: 1) neuroimaging performed to confirm the absence of infarct and 2) no exclusion criteria listed in Table 1. From the initial sample of patients, 54 TIA patients met inclusion criteria for this study. Further, all TIA patients were adjudicated by a neurologist (coauthor AK Adcock) using the American Heart Association definition of TIA - “A brief episode of focal neurological dysfunction caused by transient focal brain or retinal ischemia, with clinical symptoms typically lasting less than one hour but up to 24 hours, and without evidence of acute infarction on neuroimaging." In addition to this definition, the study neurologist also performed a detailed review of the patient's history and presentation to identify other "red flags" for TIA mimics, such as signs of classic migraine, non-focal symptoms, history of stereotyped focal spells, or any other signs that would decrease the probability that the current episode represented a TIA. In addition, we matched a sample of 54 mild AIS patients. Mild AIS patients had to meet several criteria for inclusion: 1) MRI to confirm the presence of infarct, 2) AIS of anterior circulation (anterior or middle cerebral arteries), 3) NIHSS < 5 upon presentation, and 4) no exclusion criteria listed in Table 1. From the initial sample of patients, 54 mild AIS patients met the inclusion criteria for this study. Lastly, the remaining patients who presented to the emergency department and were suspected to be AIS or TIA, but later discharged with a diagnosis of headache, migraine, or syncope, were used as an ischemic mimic group for comparison (n = 21). There have been a number of conditions that have been considered stroke-mimicking, including but not limited to, seizure, brain tumors, migraine, headache, and syncope. In our sample, stroke mimics were identified as patients who were ruled out as AIS by the absence of infarct on neuroimaging and the presence of additional “red flags” for mimics, such as stereotyped focal spells over years, classic migraine, non-focal symptoms, or any other medical diagnoses that are more often associated with mimic rather than TIA. Stroke mimics in this study were not selected or excluded on the basis of the mimicking condition but rather the headache, migraine, or syncope which were the only discharge diagnoses present in our mimic population.

**Table 1:** Study Exclusion Criteria NIHSS: National Institutes of Health Stroke Scale; tPA: tissue plasminogen activator

Study Exclusion Criteria
Age < 50 or age > 80 years
Unknown time of symptom onset
Symptom onset > 24 hours of presentation
Received tissue plasminogen activator (tPA)
Recent hospital admission (within 14 days)
Known infection within 14 days of presentation or sign of infection upon presentation
Use of immunosuppressant drugs within 14 days of presentation
NIHSS > 5 on admission

The following data were collected from medical records from all subjects: past medical history and complete blood cell count with differential (CBC-DIFF). For CBC-DIFF, the absolute count and percentage of the following immune measures were collected: WBC, neutrophil, lymphocyte, monocyte, and platelet. NLR was defined as neutrophil percent divided by lymphocyte percent.

Statistical analyses were performed using the IBM Statistical Package for Social Sciences (SPSS) Statistics® (Version 26) software (IBM SPSS Statistics for Windows, Armonk, NY), and p-values < 0.05 were considered statistically significant. Grubb’s Outlier Test was used to detect any significant outliers in all study variables and were removed prior to analysis. For each continuous variable, a Shapiro-Wilk test was used to test for normality. An independent-samples T-test or one-way analysis of variance (ANOVA) was used to detect mean differences for normally distributed immune parameters; otherwise, a Mann-Whitney U test or Kruskal-Wallis one-way ANOVA was performed. Correction for multiple testing was performed for one-way ANOVA using the Bonferroni correction, and adjusted p-values < 0.05 were considered statistically significant. For all comparisons, logistic regression analysis was used to assess for the effect of confounding cerebrovascular risk factors, such as age, gender, and cardiovascular health, for each study variable. For statistically significant predictors identified by logistic regression, a receiver operating curve (ROC) was used to determine the sensitivity and specificity of the predictor variable.

## Results

The baseline characteristics of each group of the study population are summarized in Table 2. Despite several significant differences across all three groups, mild AIS and TIA groups differed only on the basis of sex and baseline NIHSS. There was a significantly higher proportion of females in the TIA compared to the mild AIS group (p = .009), and baseline median NIHSS was significantly lower in TIA compared to mild AIS (P < .0001). In addition, there was a significant difference in the proportion of patients with NIHSS = 0 between all three groups (P < .0001) and between mild AIS and TIA.

**Table 2:** Study Population - Mild AIS, TIA, and Mimic AIS: acute ischemic stroke; IQR: interquartile range; NIHSS: National Institutes of Health Stroke Scale; IQR: interquartile range; SD: standard deviation; TIA: transient ischemic attack

	Mild AIS (n = 54)	TIA (n = 54)	Mimic (n = 21)	p-value
Age (years ± SD)	70 ± 10	72 ± 12	62 ± 13	.007
Sex (% female)	33	63	62	.015
Heart disease (%)	33	46	14	.032
Hypertension (%)	95	89	67	.010
Diabetes (%)	31	37	24	.524
Hyperlipidemia (%)	61	55	43	.406
Atrial fibrillation (%)	19	11	5	.245
Smoking (%)	22	30	19	.562
Prior stroke/TIA (%)	22	19	19	.907
Baseline NIHSS (median (IQR))	2 (3)	0 (1)	0 (2)	< .0001
Baseline NIHSS = 0 (%)	11	59	57	< .0001

Of all the immune parameters assessed in this study, mild AIS, TIA, and mimic only significantly differed on the basis of neutrophils (Table 3). Despite the lack of a significant difference in the absolute neutrophil count or neutrophil percentage, there was an unadjusted significant difference in the proportion of patients with a neutrophil percentage > 60 (above normal range) (p = .005). There was not a significant unadjusted difference in neutrophil percentage > 60 between TIA and mimic (57 vs. 57%, p = 1.000); however, the proportion of patients with neutrophil percentage > 60% was significantly higher in mild AIS group (89%) compared to both TIA (p = .002) and mimic (p = .010) groups. Further, when AIS and TIA are combined to form a single vascular group, there was no unadjusted difference in neutrophil percentage > 60 between the vascular groups (AIS + TIA) and the mimic (70 vs. 57%, p = .306).

**Table 3:** Immune Parameters - Mild AIS, TIA, and Mimic AIS: acute ischemic stroke; IQR: interquartile range; NIHSS: National Institutes of Health Stroke Scale; NLR: neutrophil-lymphocyte ratio; PLT-1: platelet; TIA: transient ischemic attack; WBC: white blood cell

	Mild AIS (n = 54)	TIA (n = 54)	Mimic (n = 21)	P-value
WBC count (median (IQR))	8.6 (2.5)	7.9 (3.2)	7.8 (3.5)	.589
> 11 x 10^3^ WBC/uL	11	6	14	.420
Neutrophil count (median (IQR))	5.6 (2.6)	4.9 (2.2)	4.9 (3.2)	.112
Neutrophil (%) (median (IQR))	66 (18)	62 (15)	61 (17)	.240
> 60% neutrophil (%)	89	57	57	.005
Lymphocyte count (median (IQR))	1.4 (0.8)	1.8 (1)	2.2 (1.3)	.063
Lymphocyte (%) (median (IQR))	22 (11)	25 (14)	27 (16)	.333
< 20% lymphocyte (%)	36	33	33	.959
NLR (median (IQR))	3 (2.9)	2.6 (2.3)	2.3 (2.7)	.182
Monocyte count (median (IQR))	0.6 (0.3)	0.6 (0.3)	0.6 (0.2)	.943
Monocyte (%) (median (IQR))	7 (3)	9 (4)	8 (4)	.336
> 8% monocyte (%)	36	52	48	.336
Platelet (PLT-I) count (median (IQR))	200 (50)	217 (65)	203 (52)	.542

To assess the ability of neutrophil percentage > 60 to predict mild AIS vs. TIA, a stepwise logistic regression model was generated, also entering age, sex, cerebrovascular risk factors, and baseline NIHSS into the model. Following the stepwise regression, age and all cerebrovascular risk factors were removed from the model, and female sex (OR = 3.2, 95% CI (1.1 - 9.1), p = .026), baseline NIHSS (OR = 8, 95% CI (2.3 - 27.8), p = .001), and neutrophil percentage > 60 (OR = 5.3 95% CI (1.1 - 26.3), p = .041) remained in the model as significant predictors of mild AIS.

**Figure 1:**
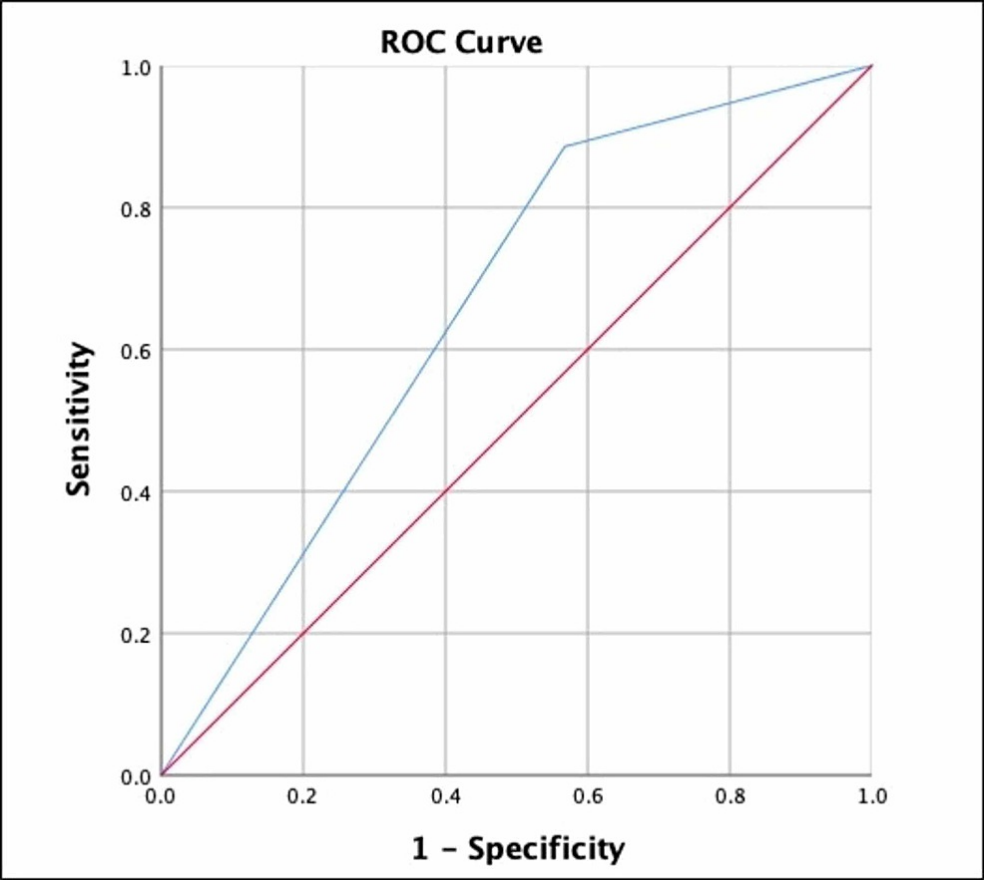
Receiver operating curve (ROC) - neutrophil percentage > 60 for prediction of mild acute ischemic stroke (AIS)

The sensitivity and specificity of the neutrophil percentage > 60 for the prediction of mild AIS were determined by interpretation of ROC, using the predicted probabilities from binary logistic regression (Figure 1). The neutrophil percentage > 60 predicted mild AIS with 88.9% sensitivity and 43.2% sensitivity (area under the curve (AUC) = 0.659, 95% CI (0.555 - 0.763), p = .007).

## Discussion

Overall, our main finding was that mild AIS patients were 5.3 times more likely than TIA patients to have a neutrophil percentage above the normal range. Thus, clinicians may utilize a neutrophil percentage as an additional piece of information that may aid in AIS diagnosis and subsequent management. We found that the proportion of patients with a neutrophil percentage above the normal range (60%) was significantly higher in mild AIS (89%) compared to both TIA and mimic. After adjustment for confounding variables using stepwise logistic regression, a neutrophil percentage > 60 remained in the model as a significant predictor of mild AIS, with mild AIS patients 5.3 times more likely than TIA cases to have a neutrophil percentage above the normal range. There was not a significant difference in neutrophil percentage > 60 between TIA and mimic. Overall, this result indicates that neutrophil percentage above the normal range may be a differential biomarker between mild AIS and TIA; however, it cannot be used to accurately differentiate TIA and mimic.

Our results are not unexpected, given that previous studies have suggested that neutrophils are elevated in AIS compared to TIA [5, 7]. Neutrophils have been traditionally viewed as harmful following a stroke, in both preclinical and clinical models. In clinical models, increased neutrophil counts within 24 hours of AIS are associated with larger infarct volumes and worse functional outcomes [8-14]. Based on our previous study, we hypothesize that there is a certain degree of peripheral neutrophil response that is proportional to the amount of brain damage or infarct volume, and both a lack of or excessive neutrophil response may be detrimental [14]. Given that the neutrophil response to AIS is correlated with the severity of ischemia, while the role of neutrophils in TIA is unknown, it may be rationalized that there is a lower degree of neutrophil activation and migration to the brain in TIA compared to AIS, due to more transient hypoxia/ischemia which resolves without permanent infarct. Despite the lack of a significant difference in the absolute neutrophil count between mild AIS and TIA, the elevated neutrophil count in AIS as compared with TIA, as well as the significantly higher proportion of AIS patients with a neutrophil percentage above the normal range, may support this hypothesis. Further, there was no difference in neutrophils between TIA and mimic. Given that stroke mimic represents a variety of diagnoses, including headache, migraine, syncope, etc., this group is highly variable on many parameters, including the immune response. Given the variability of the diagnosis and presence of cerebrovascular risk factors in the stroke mimic group, the role of immune cells in this group cannot be accurately ascertained or generalized.

Our study is novel for several reasons. First, this is the first study to include mild AIS, TIA, and mimic for comparison. No previous study has previously utilized a stroke mimic group. Second, in all prior studies, the AIS group was not limited to mild AIS (NIHSS < 5). In many cases, the differential diagnosis between moderate to severe AIS vs. TIA and mimic may not be as nuanced as compared to mild AIS patients, so while the results of previous studies have yielded some helpful information, they did not provide information on a more clinically relevant diagnosis. Lastly, many previous studies have combined AIS and TIA patients together to form a single group for comparison versus control patients. However, from both a pathophysiological and clinical management perspective, it is reasonable that AIS and TIA should also be considered separately, as we have done in this study.

There are several limitations of our study that should be addressed. The first was discussed previously but is related to the relatively small total sample size utilized in this study. Although as mentioned previously, while the study sample was sufficient to detect significant differences between mild AIS, TIA, and mimic, the relatively low sample size leads to the high variability and large confidence intervals for the described odds ratios. Similarly, our sample size limited our ability to address the influence of confounding variables on our results. For example, while patients taking immunosuppressant drugs or patients known to have an infection within 14 days of admission were excluded from our study, a person's inflammatory state and peripheral leukocyte counts can be influenced by a large number of factors, including comorbidities and medication use. Therefore, this complexity could not be comprehensively accounted for or controlled for in this study; however, a larger sample size would allow for the addition of a greater number of potentially confounding variables into regression models during analysis. Second, given that TIA is a clinical diagnosis and imaging can only rule out AIS rather than the rule in TIA, our TIA group represents our institutional vascular neurologist’s diagnosis only. Similarly, stroke mimics can often be diagnosed as TIA in the absence of any other definitive cause of symptoms; however, the definition of TIA and mimic is not unique to our study, but a challenge to the field. In this study, the only diagnoses which characterized the stroke mimic groups were headaches/migraines or syncope, but our sample did not capture the multitude of diagnoses that are considered to be stroke-mimicking conditions. As such, our study suggests that patients with headache/migraine or syncope cannot be differentiated from TIA based on leukocyte biomarkers. Thus, a larger sample, including patients with a broader representation of stroke mimicking conditions, may yield different results.

## Conclusions

Overall, our results suggest that clinicians may utilize the neutrophil percentage as an additional piece of information that may aid in their diagnosis of mild AIS versus TIA. While the accuracy of this prediction is currently unknown, the neutrophil percentage is an objective measure that may reduce the subjective diagnosis of mild AIS from conditions with similar symptoms and presentation, but where an alternative treatment strategy may be beneficial. For example, it may assist in the identification of mild AIS patients who may benefit from treatment with tPA versus those who likely will not. Lastly, while differential diagnosis between mild AIS and TIA (as we have described here) is useful, confirmation of AIS in cases of mild AIS patients whose initial CT imaging was negative for stroke is a topic that may have even greater utility to neurologists. Future studies should expand the sample included in this study to divide CT- positive mild AIS and CT-negative mild AIS into separate groups to determine if leukocyte biomarkers can differentiate CT-negative mild AIS from TIA.

## References

[1] Coutts SB (2017). Diagnosis and management of transient ischemic attack. Continuum (Minneap Minn).

[2] Powers WJ, Rabinstein AA, Ackerson T (2019). Guidelines for the early management of patients with acute ischemic stroke: 2019 update to the 2018 guidelines for the early management of acute ischemic stroke: a guideline for healthcare professionals from the American Heart Association/American Stroke Association. Stroke.

[3] Ross AM, Hurn P, Perrin N, Wood L, Carlini W, Potempa K (2007). Evidence of the peripheral inflammatory response in patients with transient ischemic attack. J Stroke Cerebrovasc Dis.

[4] Lim HH, Jeong IH, An GD (2019). Early prediction of severity in acute ischemic stroke and transient ischemic attack using platelet parameters and neutrophil-to-lymphocyte ratio. J Clin Lab Anal.

[5] Gökhan S, Ozhasenekler A, Durgun HM, Akil E, Ustündag M, Orak M (2013). Neutrophil lymphocyte ratios in stroke subtypes and transient ischemic attack. Eur Rev Med Pharmacol Sci.

[6] Celikbilek A, Ismailogullari S, Zararsiz G (2014). Neutrophil to lymphocyte ratio predicts poor prognosis in ischemic cerebrovascular disease. J Clin Lab Anal.

[7] Armstrong CWL, Bosio E, Neil C, Brown SGA, Hankey GJ, Fatovich DM (2017). Distinct inflammatory responses differentiate cerebral infarct from transient ischaemic attack. J Clin Neurosci.

[8] Ortolano F, Maffia P, Dever G (2010). Advances in imaging of new targets for pharmacological intervention in stroke: Real-time tracking of T-cells in the ischaemic brain. Br J Pharmacol.

[9] Luo Y, Xia L-X, Li Z-L, Pi D-F, Tan X-P, Tu Q (2020). Early neutrophil-to-lymphocyte ratio is a prognostic marker in acute minor stroke or transient ischemic attack. Acta Neurol Belg.

[10] Pillay J, Ramakers BP, Kamp VM (2010). Functional heterogeneity and differential priming of circulating neutrophils in human experimental endotoxemia. J Leukoc Biol.

[11] Hug A, Dalpke A, Wieczorek N (2009). Infarct volume is a major determiner of post-stroke immune cell function and susceptibility to infection. Stroke.

[12] Stowe AM, Adair-Kirk TL, Gonzales ER, Perez RS, Shah AR, Park TS, Gidday JM (2009). Neutrophil elastase and neurovascular injury following focal stroke and reperfusion. Neurobiol Dis.

[13] Gelderblom M, Leypoldt F, Steinbach K (2009). Temporal and spatial dynamics of cerebral immune cell accumulation in stroke. Stroke.

[14] Petrone AB, Eisenman RD, Steele KN, Mosmiller LT, Urhie O, Zdilla MJ (2019). Temporal dynamics of peripheral neutrophil and lymphocytes following acute ischemic stroke. Neurol Sci.

